# Prevalence of Obesity and Overweight among Adults in the Middle East Countries from 2000 to 2020: A Systematic Review and Meta-Analysis

**DOI:** 10.1155/2022/8074837

**Published:** 2022-02-03

**Authors:** Hassan Okati-Aliabad, Alireza Ansari-Moghaddam, Shiva Kargar, Neda Jabbari

**Affiliations:** ^1^Health Promotion Research Center, Zahedan University of Medical Sciences, Zahedan, Iran; ^2^MSc of Epidemiology, Gerash University of Medical Sciences, Gerash, Iran; ^3^Department of Environment Health Engineering, School of Health, Zahedan University of Medical Sciences, Zahedan, Iran

## Abstract

**Background:**

Obesity has become a significant public health issue worldwide, and it is a major risk factor for many noncommunicable diseases. This systematic review aimed to identify the prevalence of obesity and overweight in the Middle East region and different countries in this region.

**Materials and Methods:**

PubMed, Google Scholar, and MEDLINE databases were searched from 2000–2020 to identify relevant studies in the Middle East area. The survey was carried out using combinations of Medical Subject Headings (Mesh) keywords like “body mass index”, “obesity”, “overweight”, “prevalence”, “Middle-East”, and “Countries in the Middle East area”. Analysis of the data was done using STATA-14, and a random-effects model was used to estimate the pooled prevalence.

**Results:**

A total of 101 studies with 698905 participants have been identified that met inclusion criteria for this meta-analysis. The pooled estimates of the prevalence of obesity and overweight in the Middle East area were 21.17 (95% CI: 17.05–26.29) and 33.14 (95% CI: 26.87–40.87), respectively. The findings showed that obesity prevalence increased with age so that the highest prevalence of obesity and overweight was observed in people >40 years old. Obesity prevalence in the Middle East area remained steady between 2000–2006 and 2014–2020 (23%). During these time intervals, the prevalence of overweight decreased from 34.83 (95% CI: 32.40–37.45) to 32.85 (95% CI: 31.39–34.38).

**Conclusions:**

Despite the relative stabilization of the overweight and obesity trend in the Middle East, current interventions to combat the overweight epidemic need to be maintained and strengthened because the prevalence of overweight and obesity in this region is still very high. The prevalence of obesity increases with age so that people over 40 have the highest percentage of obesity and overweight. Therefore, implementing intervention programs to prevent and control obesity and overweight in the Middle East is essential.

## 1. Introduction

Obesity and overweight are health problems that indicate excessive and abnormal accumulation of body fat and lead to adverse health effects [1]. Epidemiological studies have identified obesity and overweight as risk factors for several diseases, including diabetes, various cancers, cardiovascular disease, and hypertension [2].

The increasing prevalence of high BMI and its resulting mortality threaten people's health in many countries. In addition, it causes destructive health effects and financial burden on people and society [3, 4]. The leading causes of the increase in obesity and overweight in the Eastern Mediterranean (EMRO) are lifestyle changes, including unhealthy eating habits, physical inactivity, and cultural, social, and economic changes [5, 6]. On the other hand, using a plant-based diet and physical activity in daily life reduces the risk of obesity [7]. Kuwait, Qatar, and Libya, the three EMRO countries, were among the top ten countries with the highest prevalence of obesity in the world in 2013 [8].

The body mass index (BMI) is a simple index to classify overweight and obesity in adults and is defined as weight in kg/height in m^2^. Individuals with a BMI ≥30 kg/m^2^ are considered obese, and individuals with a BMI between 25 and 29.9 kg/m^2^ are considered overweight [9]. Studies show that with age, BMI increases, which is more common in women than men [10].

Up-to-date information on the level and trend of overweight and obesity is needed to prioritize measures to prevent and control weight gain and obesity by health policymakers. Therefore, this systematic review aims to estimate the prevalence of obesity and overweight in general and based on countries in the Middle East. The study also evaluated the attributable risk of obesity-related cardiovascular disease populations in the Middle East.

## 2. Method

### 2.1. Search Strategy

Preferred Reporting Items for Systematic Reviews and Meta-Analyses (PRISMA) standards were used when conducting this systematic review [11]. A literature search was performed in the online database including Google Scholar, PubMed, and MEDLINE to find the relevant article published between 2000 and 2020. The investigation was done using keyword combinations Medical Subject Headings (Mesh) such as “body mass index”, “obesity”, “overweight”, “prevalence”, “Middle-East”, and “Countries in the Middle East area”. Two authors worked separately on the literature search.

### 2.2. Inclusion and Exclusion Criteria

The following are the criteria for including articles in the meta-analysis:

1. Studies have defined a BMI of ≥30 kg/m^2^ as obesity and a BMI of 25–29.9 kg/ as overweight
2. The classification of overweight and obese people was clearly defined
3. Cross-sectional population-based studies were performed between 2000 and 2020 that reported the prevalence of obesity and overweight
4. Adults over the age of 15 were eligible to participate in the studies

Studies were excluded from the meta-analysis if they were not published in English and if they focused on children and adolescents and populations with specific conditions, such as hypertension, diabetes, and cancer. In addition, studies that provided the only frequency of obesity and overweight, with no data to calculate the 95% confidence interval and mixed reporting of obesity and overweight were excluded.

## 3. Study Selection and Data Extraction

### 3.1. Data Extraction

All articles identified in databases were screened based on keyword, title, and abstract by two researchers independently. Then, relevant articles were assessed, and data extraction was done from the eligible articles and information stored into Microsoft Excel using a checklist created by the researcher.

Data extracted for study characteristics contained the following items: names of authors, year of publication, sample size, gender, age, study setting (country, urban/rural), the prevalence of obesity, overweight, and body mass index mean and its 95% confidence interval. Some studies, however, did not report confidence intervals. As a result, the following equation was used to calculate the relevant confidence intervals for each point estimation:

(1) $$\begin{array}{c} p+\frac{z^{2\alpha /2}}{2n}\pm z\frac{\sqrt{p\left(1-p\right)\pm z^{2\alpha /2}/4n}}{\left(1+z^{2\alpha /2}/n\right)}. \end{array}$$

### 3.2. Statistical Analysis

The random-effects models were used to generate pooled estimates. I-square and *Q* figures were also used to look at potential sources of heterogeneity.

The population attributable risks for cardiovascular diseases such as coronary heart disease (CHD), heart failure (HF), and atrial fibrillation (AF) associated with obesity were calculated by prevalence estimates of the obesity in this meta-analysis and the equation: PAR = *P* (RR − 1)/*P* (RR − 1) +. The RR was obtained from previously published recent meta-analyses that assessed the association between obesity and the disease listed above.

Therefore, the relative risk (RR) and 95% CI for atrial fibrillation (AF) were considered 1.51 (1.35–1.68) [12] and odd ratio (OR) and 95% CI for the association between obesity and coronary heart disease (CHD) and heart failure (HF) were considered 1.20 (1.02–1.41) and 1.62 (1.32–1.99), respectively [13, 14].

## 4. Result

### 4.1. Selection of Study and Characteristics

**Figure 1:**
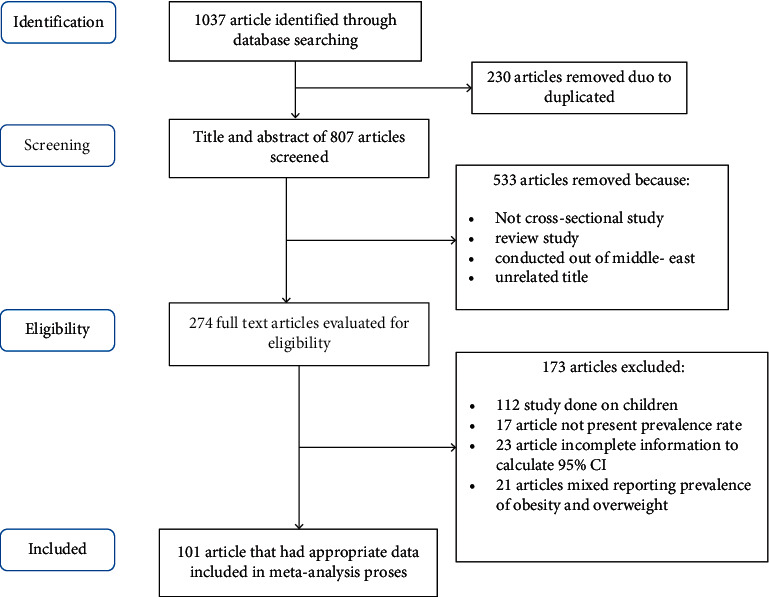
Flowchart of the study selection process.

In the primary search, 1037 articles were identified from databases, of which 230 duplicate articles were excluded. In the first phase (assessing title and abstract), 533 articles were removed due to not being a cross-sectional study design, unrelated title, out of the Middle East scope and review article nature. Finally, 274 articles were assessed in full text; of these, 101 articles met the inclusion criteria in this systematic review and meta-analysis. The flowchart of the study selection process and the frequency of factors for exclusion are outlined in Figure 1.

The studies were performed in 17 Middle East countries: Turkey (16 reports), Iran (11), Kuwait (9), Israel (2), Saudi Arabia (11), Oman (4), Palestine (6), Yemen (1), United Arab Emirates (5), Syria (2), Lebanon (6), Iraq (7), Cyprus (2), Bahrain (2), Jordan (8), and Egypt (8).

A total of 698905 participants aged >15 years were included in this systematic review. Studies had a range of sample sizes from 2500 to 257555. The articles were published between 2000 and 2020, including 18 articles during 2000–2006, 40 articles during 2007–2013, and 43 articles during 2014–2020. Moreover, four studies assessed the prevalence of obesity and overweight on only men and thirteen studies on only women.  Table 1 summarizes the characteristics of the articles that were used in the study.

### 4.2. Prevalence of Overweight and Obesity

**Figure 2:**
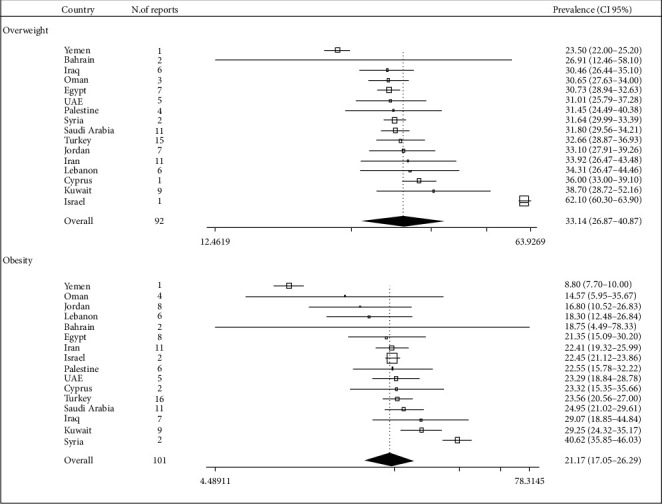
Overall prevalence of overweight and obesity in Middle East countries.

Overall, the pooled estimates of the prevalence of obesity and overweight in the Middle East countries were 21.17 (95% CI: 17.05–26.29) and 33.14 (95% CI: 26.87–40.87), respectively (Figure 2). However, some heterogeneity was observed between the results of the studies (*p* < 0.001). The range of prevalence of obesity in the Middle East region was between 40.62 (35.85–46.03) in Syria and 8.80 (95% CI: 7.70–10.00) in Yemen. Also, the range of prevalence of overweight among adults in the Middle East region was between 62.10 (95% CI: 60.30–63.90) in Israel and 23.50 (95% CI: 22.00–25.20) in Yemen.

Based on results of sex-specific subgroup analyses, the prevalence of obesity was significantly higher in women, 25.40 (95% CI: 23.66–27.27), than in men, 19.86 (95% CI: 17.60–22.40) (*p*=0.001). In contrast, men were more likely to be overweight than women, with a prevalence of 37.80 (95% CI: 36.20–39.47) compared to 31.24 (95% CI: 29.96–32.57) (*p* < 0.001).

For residency-specific subgroup analyses, although the rural population had a higher prevalence of obesity and a lower prevalence of overweight than the urban population, it was not statistically significant (*p*=0.59, *p*=0.77). The findings of age-specific subgroup studies revealed that obesity increased with age, peaking in the 50–59 and 60–69 age ranges. In addition, the 40–49 and 60–69 age groups had the highest prevalence of overweight (Table 2).

### 4.3. Time Trends in Obesity and Overweight by Country and Gender

Table 3 depicts the prevalence of obesity and overweight in the Middle Eastern countries from 2000 to 2020. From 2000 to 2006, the highest prevalence of obesity was in Saudi Arabia, 39.6 (95% CI: 37.9–41.3), and Syria, 38.2 (95% CI: 36.0–40.3). Moreover, from 2014 to 2020, the highest prevalence of obesity was in Oman, 67.81 (95% CI: 65.22–70.51), and Syria, 43.4 (95% CI: 40.2–46.6). Concerning the prevalence of overweight, from 2000 to 2006, the highest prevalence was observed in Kuwait, 44.85 (95% CI: 38.74–51.93), and Iran, 43.3 (95% CI: 37.6–49.1). Despite this, from 2014 to 2020, the highest prevalence was in Jordan, 39.94 (95% CI: 33.98–46.95), and the United Arab Emirates, 39.81 (95% CI: 33.66–47.08).

Overall, in the Middle East region, obesity prevalence remained stable from 2000 to 2006 and 2014 to 2020, with an average prevalence of 23 percent. However, the prevalence of overweight decreased from 34.83 (95% CI: 32.40–37.45) to 32.85 (95% CI: 31.39–34.38) during these time intervals.

The sex-specific subgroup prevalence showed that in women, the prevalence of obesity and overweight decreased from 26.62 (95% CI: 22.93–30.90) and 32.30 (95% CI: 29.84–34.96) during 2000 to 2006 to 23.15 (95% CI: 20.85–25.70) and 32.85 (95% CI: 31.39–34.38) during 2014 to 2020, respectively.

The prevalence of obesity in men increased from 20.08 (95% CI: 16.24–24.82) from 2000 to 2006 to 23.48 (95% CI: 20.26–27.20) from 2014 to 2020. However, the overweight prevalence was stable at these periods (39%).

### 4.4. Population Attributable Risk of Cardiovascular Disease for Obesity

Table 4 presents Population Attributable Risk (PAR) for cardiovascular disease, including coronary heart disease (CHD), heart failure (HF), and atrial fibrillation (AF). Population Attributable Risk (PAR) for cardiovascular disease was ranged from 0.3 to 19.8% by countries and about 11% of HF, 4% of CHD, and 9% of AF were related to obesity in more countries. The highest PAR was observed for heart failure (HF), of which nearly 11.5% of HF was attributed to obesity. Also, the cardiovascular disease burden related to obesity in Syria, Kuwait, and Iraq was higher than that in other countries due to the high prevalence of obesity in these countries. The fraction of cardiovascular disease attributable to obesity ranged from 3.6 to 10.5% in males and 4.7 to 13.4% in females.

## 5. Discussion

The results of this systematic review showed that the prevalence of overweight and obesity in the Middle East is 23.5–62.1 and 14.5–40.6, respectively. The difference in socioeconomic status and lifestyle between countries can explain this difference. In this study, the highest prevalence of obesity and overweight was in Kuwait, Syria, and Israel. Lifestyle changes over the past few years, including the use of Arabic diets (high-calorie and fatty foods such as fast foods), alcohol consumption, and reduced physical activity, may explain the high prevalence of noncommunicable diseases, including obesity [115].

Moreover, some studies have shown that the high prevalence of overweight and obesity in deprived sparsely populated groups is partly due to the low quality of their diet [116]. In this study, the lowest prevalence of obesity and overweight was in Yemen, which could be due to the low number of reports of obesity and the lack of new studies.

In this study, the prevalence of obesity was higher in women than men. This result is similar to the study in Spain [10] and contrasts with the study in Turkey [84]. This difference can be partly due to multiple births in women, hormonal differences between men and women, and sedentary lifestyle in women because most women are housewives or have jobs with less physical activity [117]. Studies have also shown that the prevalence of obesity in married people is increasing, which obviously puts women at even greater risk of obesity [118, 119].

Previous studies have shown that aging is strongly associated with the prevalence of obesity, and in general, the prevalence of obesity increases until age 70 and then begins to decrease [18, 120]. The present study results also show the natural pattern of obesity increase with age, at least up to 69–60 years, and the highest prevalence of obesity and overweight was seen in people over 40 years. It is thought that the decline in the prevalence of obesity in people over the age of 70 is partly due to a lower survival rate in obese people and a decrease in physical activity with increasing age in men and women. In addition, menopausal women are more prone to weight gain from 45 years [76, 121].

The present study showed that the prevalence of obesity and overweight in the Middle East in the last two decades had been almost a steady trend. However, the prevalence of obesity and overweight is at a high level. Evidence shows that the trend in mean BMI in northwestern European countries and high-income English-speaking regions and Asia-Pacific is flat for both sexes [122].

Furthermore, the results obtained from the Middle East region countries indicate a fundamental difference between the current level and trend of overweight and obesity between countries. In many countries, the prevalence of obesity and overweight has significantly decreased (Table 4). Another study showed that in many European countries, the prevalence of obesity and overweight in children has also stabilized [116]. While the prevalence of overweight and obesity seems to stabilize and even decline at different levels in different countries, it is still an important public health issue. Increasing public awareness of the effects of obesity and interventions related to daily physical activity and healthy diets have helped stabilize obesity [116, 123].

The present study showed that 4% of CAD, 11% of HF, and 9% of AF in the Middle East could be attributed to obesity. In general, approximately 8% of cardiovascular diseases in the region is related to obesity. Due to the high prevalence of obesity in people over 40 years of age, the risk of developing the disease in this age group increases. Previous studies have shown a link between obesity and cardiovascular disease [124–126]. Therefore, having a healthy lifestyle that includes healthy nutrition and adequate physical activity can significantly prevent obesity and its complications such as cardiovascular disease [59].

## 6. Conclusion

This meta-analysis showed that although the prevalence of obesity and overweight has been almost constant in the Middle East over the past two decades, the prevalence of obesity is significantly higher. In addition, the high prevalence of obesity and overweight in people over 40 years of age and the increasing trend of obesity with increasing age is a concern that should be considered by providers of intervention programs in the region. The results also showed that approximately 8% of cardiovascular diseases in the Middle East could be attributed to obesity. Therefore, obesity is a risk factor for CVD, and the necessary interventions to prevent obesity and its complications are essential.

## 7. Limitations

This study had limitations such as an unequal number of studies in countries, the use of different sampling methods, and differences in the age distribution of participants, which could be the source of differences in the prevalence of obesity and overweight in countries. Another limitation of this study is the lack of reports on obesity and overweight in urban and rural areas. Also, in this study, the unadjusted relative risk was used to calculate the attributable risk, while possible confounders such as blood pressure, smoking, family history of obesity, and socioeconomic status can confound RR as an indicator of the relationship between obesity and cardiovascular disease.

## Figures and Tables

**Table 1:** Summary of included studies in the meta-analysis.

Authors/year of publication	Setting		Sampling					Obesity			Overweight	
	Country	Rural/urban	Method	Age	N.Men	N. female	N. total	Sex/setting	Prevalence	95% CI	Prevalence	95% CI
Gunaid et al., 2012 [15]	Yemen	U	Random sample	≥20	919	1581	2500	FM	8.8	7.7–10	23.5	22–25.2
								M	2.5	1.6–3.7	18	15.5–20.4
								F	12.5	10.8–14.1	26.8	24.6–28.9
Janghorbani et al., 2007 [16]	Iran	—	Stratified probability cluster	15–65	45,082	44,322	89,404	FM	17.6	17.3–17.8	32.2	31.9–32.5
								M	10.9	10.5–11	32	31.5–32.4
								F	24.5	24–24.8	32.4	32.0–32.9
Kelishadi et al. 2007 [17]	Iran	R, U	—	15–64	45113	44344	89 532	FM	28.6	28.3–28.9	10.8	10.6–11
Djalalinia et al. 2020 [18]	Iran	R, U	Systematic random sampling cluster	≥18	14080	15,044	29,124	FM	22.7	22.2–23.2	36.6	36.0–37.1
								M	15.3	14.7–15.9	38.3	37.4–39.1
								F	29.8	29.0–30.5	35	34.2–35.8
								U	24.1	23.4–24.7	38.4	37.7–39.1
								R	19.5	18.6–20.4	32.1	31.1–33.1
Ali AL-Nooh et al. 2014 [19]	Bahrain	—	Census	39.1	641	498	1139	FM	38.7	35.7–41.5	39.7	36.8–42.5
								M	36.9	33.2–40.8	42.9	39.0–46.8
								F	40.7	36.4–45.2	23.4	19.8–27.4
Al-Ansari et al. 2000 [20]	Bahrain	U	Volunteered	17–38	238	406	644	FM	9	6.9–11.4	18.1	15.2–21.3
								M	11.1	7.6–16	17.6	13.0–23
								F	7.6	5.2–10.6	18.5	14.8–22.6
Nitzan Kaluski et al. 2007 [21]	Israeli	—	Random sample	25–64	1371	1410	2781	FM	22.8	21.2–24.4	62.1	60.3–63.9
								M	19.9	17.8–22.1	65.7	63.0–68.1
								F	25.7	23.4–28	58.8	56.1–61.3
Fraser et al., 2008 [22]	Israeli	R, U	Convenience, cluster random sample	35–64	—	—	962	FM	21.4	18.8–24.1	—	—
Shabu, 2019 [23]	Iraq	U	Multistage sampling	≥18	363	1117	1480	FM	40.9	38.2–43.3	33.4	30.9–35.8
AL-Tawil et al., 2005 [24]	Iraq	U	Convenience sample	≥18	—	200	—	F	25	19.1–31.6	39	32.2–46.1
Ali Mansour et al. 2012 [25]	Iraq	U	Simple random population-based	≥18	14,425	14,682	29,107	FM	23.8	23.3–24.2	31.3	30.7–31.8
								M	18.6	18.0–19.3	31.7	30.9–32.4
								F	28.8	28.0–29.5	30.8	30.1–31.6
Wafaa et al., 2013 [26]	Iraq	U	Probably	62.5 ± 82.1	322	178	500	FM	7.8	5.6–10.5	35.8	31.5–40.1
								M	8.1	5.3–11.6	31.6	26.6–37
								F	7.3	3.9–12.1	43.3	35.8–50.8
								U	6.6	4.4–9.2	37.1	32.6–41.7
								R	20	9.5–34.6	22.2	11.2–37
Jasim et al., 2018 [27]	Iraq	U	Randomly	—	—	—	440	F	35.2	30.7–39.8	34.5	30.1–39.1
Hayyawi et al., 2016 [28]	Iraq	U	Census	>19	—		623	FM	29.4	25.8–33.1	14.3	11.6–17.2
Al-Yasseri et al., 2019 [29]	Iraq	U	Convenience sample	50.2 ± 8.4	55	145	200	FM	81.5	75.4–86.6	—	—
								M	78.1	64.9–88.1	—	—
								F	82.7	75.6–88.5	—	—
Al-Kilani et al., 2011 [30]	Oman	U	Voluntary basis	18–25	101	101	202	FM	1.49	0.3–4.2	26.7	20.7–33.4
Louay et al., 2015 [31]	Oman	U	Census	18–24	43	183	226	FM	7.8	4.1–11.2	29.2	23.3–35.6
								M	24	11.7–38.6	39	24.9–55.5
								F	3.2	1.2–7	27	20.5–33.8
Barakat et al., 2009 [32]	Oman	R	Randomly selected	≥20	236	643	879	FM	22.9	20.1–25.7	32.3	29.2–35.5
								M	8.9	5.5–13.2	30.1	24.3–36.3
								F	28	24.5–31.6	33.1	29.4–36.9
Tengfei et al., 2020 [33]	Oman	R	Voluntarily	16–80	554	677	1231	FM	67.8	65.1–70.4	—	—
								M	24.6	20.9–28.2	—	—
								F	24.7	21.2–28.6	—	—
Abdeen et al., 2011 [34]	Palestine	R, U	Randomly	18–64	1725	1653	3378	FM	24.4	22.9–25.9	38	36.3–39.6
								M	17.5	15.7–19.3	40.3	37.9–42.6
								F	31.5	29.2–33.7	35.5	33.2–37.8
Abdul-Rahim et al., 2001 [35]	Palestine	U	—	30–65	190	295	485	FM	41	36.6–45.5	—	—
								M	30	23.5–37	—	—
								F	49	43.3–55	—	—
El Kishawi et al., 2014 [36]	Palestine	R, U	Multistage sampling	18–50	_	357	_	F	29.4	242.7–34.4	33.4	29.5–39.6
								U	31	22.1–41	26	17.7–35.7
								R	20	9.0–35.6	47.5	31.5–63.8
Stene et al., 2001 [37]	Palestine	R	—	30–65	209	269	478	FM	29.2	25.2–33.5	36.4	32.0–40.8
								M	18.8	13.6–24.6	39.7	33.0–46.6
								F	37.5	31.7–43.6	33.8	28.2–39.8
Damiri et al., 2017 [38]	Palestine	U	2 stages stratified random sampling	18–24	352	498	850	FM	5.2	3.8–7	20.9	18.2–23.8
								M	9.1	6.3–12.5	27.2	22.6–32.2
								F	2.6	1.4–4.4	16.4	13.3–20
El Kishawi et al., 2016 [39]	Palestine	U	—	18–50	_	357	_	F	29.6	25.0–34.7	_	_
Weiderpass et al., 2019 [40]	Kuwaiti	—	Random sampling	18–69	1381	2208	3589	FM	40.3	38.6–42	37	35.4–38.7
								M	36.5	33.9–39.1	42	39.3–44.6
								F	44	42.3–46.5	32.5	30.6–34.5
Al Rashdan and Al Nesef 2010 [41]	Kuwaiti	—	Random sample	20–65	918	1362	2280	FM	47.5	45.3–49.5	80.4	78.7–82
								M	39.2	36.0–42.4	_	_
								F	53	50.3–55.6	_	_
AlMajed et al., 2011 [42]	Kuwaiti	U	Randomly	17–24	173	311	484	FM	19.8	16.3–23.6	30.6	26.5–34.9
Raman et al., 2012 [43]	Kuwaiti	U	Convenience sample	≥20			432	FM	20.8	17.1–24.9	39.8	35.1–44.6
Al-Asi 2003 [44]	Kuwaiti	—	—	<40			3282	FM	27.4	25.9–28.9	47.9	46.2–49.7
Badr et al., 2012 [45]	Kuwaiti	U	A multistage cluster sampling	>50	948	1395	2443	FM	45.6	43.6–47.6	35.6	33.7–37.5
								M	30.2	27.2–33.2	45.6	42.3–48.8
								F	55.5	52.8–57.9	29.2	26.8–31.5
Alkazemi et al., 2019 [46]	Kuwaiti	U	Convenience sample	21.57 + 1.99	193	422	615	FM	15.7	12.9–18.9	22.7	19.5–26.2
								M	23.8	17.7–30.6	28.7	22.2–35.9
								F	12.1	0.9–15.7	19.9	16.0–24.1
Al-Isa, 2004 [47]	Kuwaiti	—	Systematic random sampling	>20	—	485	—	F	19.7	16.2–23.6	41.2	36.6–45.8
Zaghloul et al., 2013 [48]	Kuwaiti	—	Using stratified sampling, randomly	≥19	469	580	1049	FM	43.1	40.0–46.1	33.1	30.2–36
Adel Bakir et al., 2017 [49]	Syria	U	Randomly	18–60	—	923	—	F	43.4	40.2–46.6	31.3	28.3–34.4
Fouad, 2006 [50]	Syria	—	Stratified, cluster sampling, randomly	18–65	919	1117	2038	FM	38.2	36.0–40.3	31.8	29.8–33.9
								M	28.4	25.5–31.4	37	33.8–40.2
								F	46.2	43.3–49.2	27.6	25.0–30.3
Andreou et al., 2012 [51]	Cyprus	U	Stratified random sample	18–80	485	516	1001	FM	29	26.2–31.9	36	33.0–39.1
								M	28.8	27.0–35.4	46.9	42.2–51.3
								F	27	24.8–33.1	26	23.6–31.8
Heracldes et al., 2015 [52]	Cyprus	—	Stratifying sampling	24–65	1393	1628	3021	FM	18.8	17.4–20.2	—	—
								M	21.5	19.4–23.7	—	—
								F	16.5	17.2–21.4	—	—
Abu-Zaiton and Fawwaz 2013 [53]	Jordan	—	Multistage cluster sampling	>18	49	71	120	FM	8.3	4.0–14.7	21.67	14.6–30.11
Suleiman et al., 2009 [54]	Jordan	U	Multistage cluster sampling	17–28	428	791	1219	FM	10.1	8.5–12	28.5	26.0–31.1
								M	8.8	6.3–11.9	23.3	19.4–27.6
								F	10.8	8.7–13.2	31.3	28.1–34.7
Atoom, 2018 [55]	Jordan	—	Multistage random	16–46	570	—	—	M	16.8	13.8–20.1	36.3	32.3–40.4
Khader et al., 2009 [56]	Jordan	U	Systematic random	18–70	168	172	340	FM	30.5	25.7–35.7	33.8	28.8–39.1
Matalqah et al., 2019 [57]	Jordan	U	Convenience sampling	>18	605	310	915	FM	23	20.2–25.6	—	—
Ahmad et al., 2006 [58]	Jordan	R	Proportional sampling technique	20–25		233		F	6.8	3.9–10.9	27	21.4–33.2
Khamaiseh et al., 2015 [59]	Jordan	U	Random sampling	18–24	54	123	177	FM	14.7	9.8–20.7	49.1	41.5–56.7
								M	11.1	4.1–22.6	57.4	43.2–70.7
								F	16.2	10.2–23.9	45.5	36.5–54.7
Abu Ghazaleh and Budair 2013 [60]	Jordan	U	—	43.2	4962	3384	8346	FM	42.3	41.2–43.3	25.7	24.8–26.7
								M	52.6	50.9–54.3	33.6	32.0–35.2
								F	51.7	50.0–53.4	29.9	28.4–31.5
Alarjan et al., 2015 [61]	Jordan	R, U	Randomly	—	285	463	748	FM	11	8.8–13.4	36.9	33.4–40.4
								M	15.4	11.4–20.1	47.7	41.8–53.6
								F	8.2	5.8–11	30.2	26.0–34.6
Mowafi et al., 2013 [62]	Egypt	U	Stratified random sample	≥22	1823	1723	3993	FM	32.3	30.8–33.7	32.8	31.4–34.3
								M	23.8	21.9–25.8	41.9	39.6–44.2
								F	49.6	47.2–52	31.8	29.6–34
Abdel Sadek et al., 2016 [63]	Egypt	U	Multistage stratified random sampling	17–27			842	FM	6.6	5.0–8.5	28.8	25.8–32
Mohamed Shebl et al., 2015 [64]	Egypt	U	—	≥60	50	75	126	FM	33.3	25.1–42.2	—	—
Abdel Rahman et al., 2012 [65]	Egypt	U	Random sample	≥60	112	207	319	FM	32.2	27.1–37.7	29.4	24.5–34.8
Genena and Salama, 2017 [66]	Egypt	U	Randomly	18–26	141	257	389	FM	11.8	8.8–15.3	28.9	24.4–33.6
								M	14.1	8.8–21	33.3	25.6–41.7
								F	10.5	7.0–14.9	26.5	21.1–32.3
Farrag et al., 2015 [67]	Egypt	U	—	19.5 ± 2.0	656	1182	1838	FM	10.7	9.3–12.2	27.8	25.8–29.9
Mahfouz et al., 2006 [68]	Egypt	R	Systematically random	≥60	136	21 4	350	FM	28.3	23.6–33.3	34	29.0–39.2
								M	30.8	24.7–37.5	31.1	25.1–37.9
								F	24.2	17.3–32.3	38.2	30.0–46.9
Yount and Li, 2011 [69]	Egypt	—	—	15–49		5015		F	48.4	47.0–49.7	32.5	31.2–33.9
Sakr et al., 2016 [70]	Lebanon	U	—	16–32	140	260	400	FM	5.2	3.2–7.9	20	16.1–24.2
								M	2.1	0.4–6.1	7.1	3.4–12.7
								F	3	1.3–5.9	13	9.2–17.7
Fahs et al., 2017 [71]	Lebanon	R, U	Stratified cluster, randomly	≥45			1000	FM	29.1	26.3–32	46.1	42.9–49.2
								U	27.6	22.8–32.7	48.9	43.4–54.4
								R	30.1	26.3–33.4	45.1	40.8–48.5
Naja et al., 2011 [72]	Lebanon	R, U	—	20–55	923	1125	2048	FM	42.3	40.1–44.5	56.3	54.1–58.5
								M	24.4	21.7–27.3	65.7	62.7–68.9
								F	20.1	17.8–22.6	48.5	45.5–51.5
Yahia et al., 2008 [73]	Lebanon	U	Randomly	20 ± 1.9	96	124	220	FM	7.2	4.2–11.5	24	18.6–30.3
								M	12.5	6.6–20.8	37.5	27.8–47.9
								F	3.2	0.8–8	13.7	8.1–21.04
Haidar et al., 2016 [74]	Lebanon	U	Randomly	≥18	149	151	300	FM	27	22.0–32.4	37.6	32.1–43.4
								M	26.1	19.3–34	40.9	32.9–49.2
								F	27.8	20.8–35.6	35.7	28.1–43.9
Samhat et al., 2020 [75]	Lebanon	U	Randomly	24–45	101	206	307	FM	19.2	14.9–24	31.6	26.4–37.1
Delibasi et al., 2007 [76]	Turkey	R, U	Randomly	≥18			8674	FM	15.6	14.8–16.3	19	18.1–19.8
Erem et al., 2001 [77]	Turkey	U	Random cluster sampling	≥20	1324	1322	2646	FM	17.4	16.0–18.9	41.5	39.6–43.4
								M	10.5	8.9–12.3	46.5	43.8–49.2
								F	24.3	22–26.7	36.5	33.9–39.2
D. Yumuk et al., 2005 [78]	Turkey	U	Invited	≥20	5866	7000	12866	FM	27.4	26.6–28.1	36.2	35.3–37
								M	16.8	15.8–17.8	38.7	37.4–39.9
								F	36.2	35.1–37.4	34.1	32.9–35.2
Akbay et al., 2003 [79]	Turkey	U	Stratified random sampling	20–74	641	855	1496	FM	29.3	26.9–31.6	38.4	35.9–40.9
								M	22.3	19.1–25.7	43.2	39.3–47.1
								F	34.5	31.3–37.8	34.9	31.6–38.1
Bagriacik et al., 2009 [80]	Turkey	U	Randomly	≥20	6756	6998	13754	FM	29.5	28.7–30.2	39.5	38.7–40.4
								M	21.8	20.8–22.8	44.8	43.6–46
								F	36.9	35.7–38	34.5	33.4–35.6
Gültekin et al., 2009 [81]	Turkey	R, U	Multistage	18–65	1050	1050	2100	FM	27	25.1–29	34.4	32.4–36.5
								M	20	17.6–22.5	38	35.0–41
								F	34.1	31.2–37	30.8	28.0–33.7
Ardahan and Konal, 2019 [82]	Turkey	U	Voluntarily	51.74 ± 14.74	547	476	1023	FM	30.5	27.6–33.4	—	—
								M	24.6	21.1–28.5	—	—
								F	37.1	32.8–41.7	—	—
Arıkan et al., 2014 [83]	Turkey	R, U	Multistage stratified	>15	1015	1023	2,038	FM	24.2	22.4–26.2	34.3	32.2–36.4
Delibasi et al., 2007 [76]	Turkey	R, U	Randomly	≥18	—	—	8764	FM	15.6	14.8–16.3	19	18.1–19.8
Ustu et al., 2012 [84]	Turkey	U	Random cluster	≥18	3277	1885	5162	FM	29.5	28.2–30.7	30	28.7–31.2
								M	33.6	31.9–35.2	27.6	26.0–29.1
								F	22.3	20.4–24.2	34.1	32.0–36.3
Yabanci et al., 2010 [85]	Turkey	U	Voluntarily	18–59	527	539	1066	FM	9.7	7.9–11.6	34.8	31.9–37.7
								M	8.3	6.1–11	40.9	36.7–45.3
								F	10.9	8.4–13.8	28.7	24.9–32.7
Ahin et al., 2011 [86]	Turkey	U	Randomly	≥20	1524	—	—	M	16.8	15–18.8	38.9	36.5–41.4
Erem et al., 2004 [87]	Turkey	U	Random cluster sampling	≥20	2288	2728	5016	FM	23.5	22.3–24.7	36.8	35.4–38.1
								M	16.4	14.9–18	46.5	44.4–48.6
								F	29.4	27.6–31.1	28.6	26.9–30.3
Dinc et al., 2006 [88]	Turkey	U	Randomly	15–49	—	1602	—	F	31.9	29.6–34.3	32.2	29.9–34.6
Yalcin et al., 2004 [89]	Turkey	U	Multistage sampling	18–65	980	956	1936	FM	27.3	25.3–29.3	36.1	33.9–38.2
Ucan and Ovayolu, 2010 [90]	Turkey	U	—	≥18	749	852	1601	FM	41.8	39.4–44.3	30.5	28.2–32.8
Kerkadi et al., 2003 [91]	UAE	U	—	18–25	_	386	_	F	6.7	4.4–9.7	19.4	15.6–23.7
Kalavathy et al., 2019 [92]	UAE	_	Convenience sampling	18–77	452	92	544	FM	31.4	27.5–35.5	36.2	31.8–40.3
Hajat et al., 2012 [93]	UAE	U	—	18–75	—	—	50138	FM	35.4	34.6–35.4	31.9	31.5–32.3
								M	31.6	31.0–32.2	36.1	35.4–36.7
								F	38.3	37.8–38.9	28.8	28.2–29.3
Sulaiman et al., 2017 [94]	UAE	—	Systematic random sampling	≥18	2204	520	2724	FM	32.3	30.5–34.1	43	41.1–44.9
								M	31.3	29.4–33.3	44.7	42.6–46.8
								F	36.1	32–40.5	35.1	31.0–39.4
Sheikh-Ismail et al., 2009 [95]	UAE	U	Random sample	20–90	—	724	—	F	16	13.4–18.9	27	23.8–30.4
Alhakbany et al., 2018 [96]	Saudi Arabia	U	Multistage stratified cluster sampling	14–25	—	454	—	F	8.1	5.8–11	21.4	17.6–25.4
Al-Rethaiaa et al., 2010 [97]	Saudi Arabia	U	Randomly	14–24	357	—	—	M	15.7	12.0–19.8	21.8	17.6–26.5
Al-Baghli et al., 2008 [98]	Saudi Arabia	—	Invited	≥30	99946	95905	195874	FM	43.8	43.5–44	35.1	34.9–35.3
								M	36.1	35.8–36.4	40.3	40.0–40.6
								F	51.8	51.5–52.1	29.6	29.3–29.9
Al-Qahtani, 2019 [99]	Saudi Arabia	U	Voluntary	—	949	732	1681	FM	27.6	25.4–29.8	38.3	35.9–40.7
								M	26.2	23.1–28.8	40.2	37.1–43.4
								F	29.1	24.7–31.4	35.4	30.5–37.5
Alsaif et al., 2002 [100]	Saudi Arabia	R, U	A multistage stratified cluster sampling	30–70	1613	1648	3261	FM	39.6	37.9–41.3	36.6	35.0–38.3
								M	29.9	27.7–32.2	41.9	39.4–44.3
								F	49.1	46.7–51.5	31.5	29.3–33.8
Horaib et al., 2013 [101]	Saudi Arabia	—	Multistage stratified random	34.12 ± 7.25	—	—	10,229	FM	29	28.1–29.9	40.8	39.9–41.8
Baig et al., 2015 [102]	Saudi Arabia	U	—	22.40 ± 3.90	610	—	—	M	18.5	15.5–21.8	29.8	26.2–33.6
Al-Ghamdi et al., 2018 [103]	Saudi Arabia	R, U	Multistage sampling	≥18	381	638	1019	FM	27.5	24.8–30.4	26.6	24.0–29.5
								M	36.2	31.3–41.2	32.2	27.6–37.2
								F	22.4	19.2–25.8	23.3	20.1–26.8
Alharthi et al., 2017 [104]	Saudi Arabia	U	Convenience sampling	20–40	387	120	507	FM	29.5	25.6–33.7	36.6	32.4–41
Balgoon et al., 2019 [105]	Saudi Arabia	U	—	18–25	—	164	—	F	14	9.1–20.3	17.6	12.1–24.4
Al-Raddadi et al., 2019 [106]	Saudi Arabia	U	Stratified cluster sampling	≥18	667	752	1419	FM	35.2	32.7–37.7	32.4	29.9–34.9
								M	34.8	31.1–38.5	35	31.4–38.8
								F	35.6	32–39	30	26.7–33.4
Tabrizi et al., 2017 [107]	Iran	U	Multistage stratified cluster sampling	15–65	1368	1450	2818	FM	24	22.4–25.6	39.6	37.7–41.4
GHaderian et al., 2018 [108]	Iran	U	Random cluster sampling	≥20	1187	1388	2575	FM	26.5	25.1–28.5	39.3	37.4–41.3
								M	18.4	16.2–20.7	41	38.2–43.8
								F	34	31.5–36.5	37.9	35.4–40.5
Rezaeian and Salem 2007 [109]	Iran	U	Random sample	>30	316	440	756	FM	11.5	9.3–14	38.2	34.7–41.8
								M	3.8	1.9–6.5	37.3	31.9–42.9
								F	17	13.6–20.8	38.8	34.2–43.5
Ayatollahi and Ghoreshizadeh 2010 [110]	Iran	U	Random multistage sample	25–55	1141	1141	2282	FM	16.5	15–18.1	40.2	38.2–42.3
								M	10.5	8.8–12.5	39.2	36.2–42.1
								F	22.5	21.1–25	41.4	38.2–43.9
Nikooyeh et al., 2016 [111]	Iran	U	Randomly	20–60	114	135	249	FM	33.3	27.5–39.5	36.5	30.5–42.8
								M	25.4	17.7–34.4	38.6	29.6–48.1
								F	40	31.6–48.7	34.8	26.8–43.4
Dastgiri et al., 2006 [112]	Iran	U	Simple random	≥18	132	168	300	FM	22.4	17.0–27.6	43.3	37.6–49.1
								M	18	12.5–25.6	40.9	32.4–49.8
								F	24	18.5–31.4	45.2	37.5–53
Najafi et al., 2020 [113]	Iran	U	—	≥35	57,614	71,643	129,257	FM	30.43	30.1–30.6	40.76	40.4–41
								M	18.75	18.4–19	42.98	42.5–43.3
								F	39.83	39.4–40	38.98	38.6–39.3
Marzban et al., 2020 [114]	Iran	U	Multistage systematic sampling	20–70	395	395	790	FM	21.51	18.7–24.5	35.44	32.1–38.8
								M	31.6	27.0–36.4	52.1	47.1–57.1
								F	11.3	8.4–14.9	18.7	15.0–22.9

**Table 2:** The prevalence of obesity and overweight in the Middle East countries.

Variables	N. of studies (population)	Obesity		Test for heterogeneity	Overweight		Test for heterogeneity
		NR^*∗*^	Prevalence (95% CI)	(*p*-value)	NR^*∗*^	Prevalence (95% CI)	(*p*-value)
**Country**				*p* < 0.001			*p* < 0.001
Kuwait	9 (14174)	9	29.25 (24.32–35.17)		9	38.70 (28.72–52.16)	
Israel	2 (3743)	2	22.45 (21.12–23.86)		1	62.10 (60.30–63.90)	
Saudi Arabia	11 (215575)	11	24.95 (21.02–29.61)		11	31.80 (29.56–34.21)	
Oman	4 (2538)	4	14.57 (5.95–35.67)		3	30.65 (27.63–34.00)	
Palestine	6 (5905)	6	22.55 (15.78–32.22)		4	31.45 (24.49–40.38)	
Yemen	1 (2500)	1	8.80 (7.70–10.00)		1	23.50 (22.00–25.20)	
United Arab Emirates	5 (54516)	5	23.29 (18.84–28.78)		5	31.01 (25.79–37.28)	
Turkey	16 (71268)	16	23.56 (20.56–27.00)		15	32.66 (28.87–36.93)	
Syria	2 (2961)	2	40.62 (35.85–46.03)		2	31.64 (29.99–33.39)	
Lebanon	6 (4275)	6	18.30 (12.48–26.84)		6	34.31 (26.47–44.46)	
Iraq	7 (32550)	7	29.07 (18.85–44.84)		6	30.46 (26.44–35.10)	
Cyprus	2 (4022)	2	23.32 (15.25–35.66)		1	36.00 (33.00–39.10)	
Bahrain	2 (1783)	2	18.75 (4.49–78.33)		2	26.91 (12.46–58.10)	
Jordan	8 (12668)	8	16.80 (10.52–26.83)		7	33.10 (27.91–39.26)	
Egypt	8 (12872)	8	21.35 (15.09–30.20)		7	30.73 (28.94–32.63)	
Iran	11 (257555)	11	22.41 (19.32–25.99)		11	33.92 (26.47–43.48)	
**Total (Middle East)**	101 (698905)	101	21.17 (17.05–26.29)		92	33.14 (26.87–40.87)	
Sex							
Female	70 (361960)	70	25.40 (23.66–27.27)	*p*=0.001	63	31.24 (29.96–32.57)	*p* < 0.001
Male	62 (340723)	62	19.86 (17.60–22.40)		56	37.80 (36.20–39.47)	
Residency							
Urban	4 (21684)	4	19.89 (13.59–29.11)	*p*=0.59	4	38.89 (33.53–45.11)	*p*=0.77
Rural	4 (9337)	4	22.81 (16.27–31.96)		4	37.19 (28.41–48.69)	
Age group							
18–29	8 (17825)	8	10.46 (7.56–14.47)	*p* < 0.001	8	27.51 (21.51–35.18)	*p* < 0.001
30–39	9 (213681)	9	21.76 (17.10–27.70)		9	18.32 (18.37–23.35)	
40–49	9 (213681)	9	29.19 (23.43–36.37)		9	44.19 (37.80–51.67)	
50–59	9 (213681)	9	37.05 (31.76–43.22)		9	37.71 (32.79–43.36)	
60–69	8 (238548)	8	36.10 (32.01–40.72)		8	40.45 (35.90–45.57)	
≥70	5 (206524)	5	24.05 (18.65–31.02)		5	36.10 (33.34–39.09)	

^*∗*^NR: number report.

**Table 3:** Trends in the Prevalence of Obesity and Overweight in Middle East countries.

Country	Pooled estimate (95% CI)					
	2000–2006		2007–2013		2014–2020	
	Obesity	Overweight	Obesity	Overweight	Obesity	Overweight
	Prevalence (95% CI)	Prevalence (95% CI)	Prevalence (95% CI)	Prevalence (95% CI)	Prevalence (95% CI)	Prevalence (95% CI)
Kuwait	23.53 (17.04–32.48)	44.85 (38.74–51.93)	33.95 (28.02–41.14)	41.01 (24.89–67.58)	25.27 (10.03–63.66)	29.13 (18.05–47.02)
Israel	**NA**	**NA**	22.45 (21.12–23.86)	62.1 (60.3–63.9)	**NA**	**NA**
Saudi Arabia	39.6 (37.9–41.3)	36.6 (35.0–38.3)	35.65 (23.80–53.40)	37.82 (32.64–43.83)	20.98 (16.88–26.08)	28.10 (24.18–32.64)
Oman	**NA**	**NA**	13.79 (4.79–39.45)	31.73 (29.04–34.67)	67.81 (65.22–70.51)	29.2 (23.3–35.6)
Palestine	34.71 (24.89–48.40)	36.4 (32–40.8)	24.4 (22.9–25.9)	38.0 (36.3–39.6)	16.12 (4.15–62.62)	26.39 (16.67–41.78)
Yemen	**NA**	**NA**	8.8 (7.7–10)	23.5 (22–25.2)	**NA**	**NA**
United Arab Emirates	6.7 (4.4–9.7)	19.4 (15.6–23.7)	23.91 (10.98–52.07)	29.68 (25.25–34.90)	32.15 (30.55–33.84)	39.81 (33.66–47.08)
Turkey	25.68 (22.53–29.28)	36.86 (34.93–38.90)	21.21 (16.39–27.45)	29.70 (23.50–37.54)	27.12 (21.62–34.02)	34.3 (32.2–36.4)
Qatari						
Syria	38.2 (36.0–40.3)	31.8 (29.8–33.9)	**NA**	**NA**	43.4 (40.2–46.6)	31.3 (28.3–34.4)
Lebanon	**NA**	**NA**	17.77 (3.13–100.76)	37.08 (16.08–85.50)	17.72 (11.25–27.91)	32.64 (23.46–45.40)
Iraq	25.0 (19.1–31.6)	39.0 (32.2–46.1)	13.78 (4.62–41.11)	33.01 (29.02–37.55)	43.17 (27.26–68.39)	25.69 (16.92–39.01)
Cyprus	**NA**	**NA**	29.0 (26.2–31.9)	36.0 (33.0–39.1)	18.8 (17.4–20.2)	**NA**
Bahrain	9.0 (6.9–11.4)	18.1 (15.2–21.3)	**NA**	**NA**	38.7 (35.7–41.5)	39.7 (36.8–42.5)
Jordan	**NA**	**NA**	23.60 (10.45–53.30)	28.63 (25.02–32.78)	15.96 (11.18–22.77)	39.94 (33.98–46.95)
Egypt	28.3 (23.6–33.3)	34.0 (29.0–39.2)	37.06 (26.83–51.21)	32.54 (31.58–33.52)	12.89 (7.17–23.16)	28.21 (26.65–29.86)
Iran	22.4 (17–27.6)	43.3 (37.6–49.1)	17.74 (12.61–24.97)	27.02 (13.28–54.94)	25.98 (22.15–30.47)	38.29 (36.0–40.72)
Sex						
Female	26.62 (22.93–30.90)	32.30 (29.84–34.96)	27.20 (23.70–31.22)	33.07 (31.17–35.09)	23.68 (21.16–26.51)	28.87 (26.97–30.91)
Male	20.08 (16.24–24.82)	39.14 (36.0–42.57)	17.09 (14.0–20.87)	36.79 (34.08–39.72)	23.48 (20.26–27.20)	39.03 (37.05–41.10)
**Total (Middle East)**	**23.98 (21.24–27.08)**	**34.83 (32.40–37.45)**	**22.62 (20.18–25.35)**	**32.02 (28.56–35.89)**	**23.15 (20.85–25.70)**	**32.85 (31.39–34.38)**

**Table 4:** Population Attributable Risk for obesity by country and cardiovascular diseases.

Variables/PAR		Cardiovascular diseases		
		Coronary heart disease (CAD)	Heart failure (HF)	Atrial fibrillation (AF)
Countries	Kuwait	5.4 (0.4–12.5)	15.2 (7.1–25.7)	12.8 (7.1–19.2)
	Israel	4.2 (04–8.6)	6.5 (6.2–18.5)	10.0 (6.8–13.5)
	Saudi Arabia	4.5 (0.4–10.6)	12.9 (6.2–22.3)	10.9 (6.8–16.4)
	Oman	2.7 (0.09–12.5)	7.9 (1.5–25.7)	6.6 (1.7–19.2)
	Palestine	4.2 (0.2–11.5)	12.0 (4.5–24.0)	10.0 (4.9–17.8)
	Yemen	1.5 (0.1–3.9)	4.7 (2.1–9.0)	3.9 (2.3–6.3)
	United Arab Emirates	4.3 (0.3–10.2)	12.4 (5.4–21.7)	10.4 (5.9–15.9)
	Turkey	4.3 (0.3–9.9)	12.4 (6.0–21.0)	10.4 (6.5–15.5)
	Syria	7.4 (0.6–15.8)	19.8 (10.0–31.2)	16.9 (10.9–23.8)
	Lebanon	3.4 (0.2–9.6)	10.0 (3.6–20.4)	8.4 (4.0–15.0)
	Iraq	5.4 (0.3–15.2)	15.2 (5.4–30.3)	12.8 (5.9–23.0)
	Cyprus	4.3 (0.2–12.5)	12.4 (4.5–25.7)	10.4 (4.9–19.2)
	Bahrain	0.3 (0.07–24.2)	1.2 (1.2–43.5)	1.0 (1.3–34.6)
	Jordan	3.1 (0.1–9.5)	9.0 (3.1–20.4)	7.5 (3.3–15.0)
	Egypt	4.0 (0.2–10.9)	11.5 (4.5–22.8)	9.6 (4.9–16.9)
	Iran	4.2 (0.3–9.2)	12.0 (5.7–19.8)	10.0 (6.2–14.5)

**Sex**	Female	4.7 (0.4–9.9)	13.4 (6.8–21.0)	11.3 (7.4–15.5)
	Male	3.6 (0.3–8.2)	10.5 (5.1–16.7)	8.8 (5.6–13.0)

**Total (Middle East)**		**4.0 (0.3–9.6)**	**11.5 (5.1–20.4)**	**9.6 (5.6–15.0)**

## Data Availability

Data are available upon request.
